# CAR T cell therapy-related lumbosacral polyradiculopathy with myelitis and stiff person syndrome with response to intravenous immunoglobulin and corticosteroids in a patient with acute lymphoblastic leukemia

**DOI:** 10.1016/j.htct.2025.103932

**Published:** 2025-07-09

**Authors:** Hannah Goulart, Kevin Elmore, Stephen Scelsa, Daniel Park, Alla Keyzner, Uroosa Ibrahim

**Affiliations:** a Department of Cancer Medicine, University of Texas MD Anderson Cancer Center, Houston, USA; b Department of Neuro-oncology, Tisch Cancer Institute, Icahn School of Medicine at Mount Sinai, New York, USA; c Division of Neurology, Department of Internal Medicine, Icahn School of Medicine at Mount Sinai, New York, USA; d Stem Cell Transplantation and Cellular Therapy, Tisch Cancer Institute, Icahn School of Medicine at Mount Sinai, New York, USA

## Introduction

Chimeric antigen receptor (CAR) T cell therapy has emerged as a promising treatment for relapsed/refractory acute lymphoblastic leukemia (ALL) in children and adults. However, major toxicities can occur after CAR T cell therapy, most notably cytokine release syndrome (CRS) and immune effector cell-associated neurotoxicity syndrome (ICANS). The pathophysiology and long-lasting effects of these adverse reactions are still unknown. We report a case of lumbosacral polyradiculopathy with myelitis and stiff person syndrome (SPS) with glycine receptor antibodies after treatment with CAR T cells in a patient with ALL. To our knowledge, this is the first such report in the literature of this manifestation in a patient receiving CAR T cell therapy.

## Case

A 55-year-old female diagnosed with CD20^+^ Philadelphia chromosome-positive B-cell ALL underwent induction chemotherapy following the European Working Group on Adult ALL (EWALL) protocol using a combination of dasatinib and rituximab. The patient received a reduced intensity conditioning regimen of fludarabine and melphalan and underwent a peripheral blood stem cell transplant from a matched related donor while in first complete remission with measurable residual disease (MRD) *BCR-ABL1* by polymerase chain reaction (PCR). The patient experienced T315I mutated relapsed disease with a detectable *BCR-ABL1* PCR of 18.1521 on Day +144. There was evidence of bone marrow involvement on Day +183 and she was started on blinatumomab and ponatinib therapy. The patient received five cycles of blinatumomab followed by a donor lymphocyte infusion on Day +307.

The patient was scheduled for a second donor lymphocyte infusion but developed facial pain and swelling, prompting teeth extractions. She continued to experience facial pain and underwent a computer tomography scan of the sinuses on post-transplant Day +482, which showed ethmoid and maxillary sinus mucosal thickening and soft tissue inflammatory changes. On Day +489, she underwent maxillary antrostomy, total ethmoidectomy, and sphenoidotomy. Pathology of the resected tissue was positive for CD20^+^ *B*-ALL, consistent with extramedullary involvement of the sinuses.

**Figure 1:**
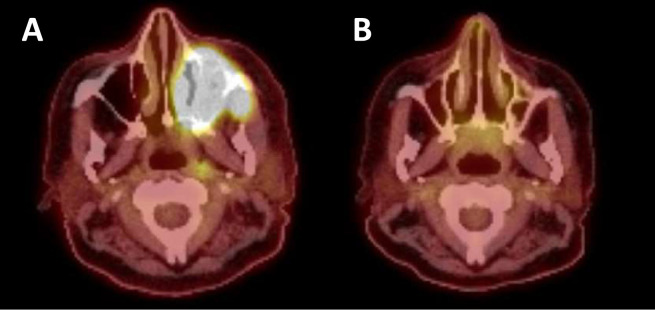
A) Positron emission tomography – computed tomography (PET-CT) scan demonstrating extramedullary disease prior to bridging and CAR T cell therapy B) PET-CT scan demonstrating response to bridging and CAR T cell therapy.

Blinatumomab and ponatinib were administered in preparation for leukapheresis, which occurred on Day +496. The patient received bridging therapy with attenuated FLAG-Ida (fludarabine, cytarabine, granulocyte colony stimulating factor, and idarubicin) resulting in MRD negative remission with a positron emission tomography (PET) scan consistent with resolution of extramedullary disease (Figure 1). The patient received brexucabtagene autoleucel (Tecartus), which was complicated by Grade 1 CRS on Day +10 and resolved with a dose of tocilizumab.

Six days after receiving CAR T cell therapy, the patient reported body aches in the upper extremities, back, and hips, which were initially thought to be related to the use of granulocyte colony stimulating factor. The pain moved to the lower back and lower extremities and was described as “electrical” and “burning.” Hydromorphone and gabapentin were given with minimal improvement in symptoms. Over the course of the ensuing three weeks the pain severely limited her ability to walk, necessitating the use of a cane. Neurologic examination showed diffuse hyperreflexia, bilateral lower extremity weakness, distal sensory loss, and wide-based unsteady gait. A total spine magnetic resonance imaging exam (MRI) performed on Day +39 was significant for a circumferential disc bulge at L5-S1 and an annular fissure, both impinging on the bilateral descending S1 nerve roots; however, no spinal cord compression, signal abnormalities, or enhancement were observed. Electromyography (EMG) on Day +52 demonstrated active denervation throughout both lower extremities with slight myopathic dysfunction in both hip flexors and right biceps. A brain MRI on Day +53 was normal. A lumbar puncture was performed on Day +55 with cerebrospinal fluid (CSF) analysis showing lymphocytic pleocytosis (white blood count: 9/µL with 86 % lymphocytes) with normal protein and glucose. CSF cytology and flow cytometry did not demonstrate any malignant cells. The presentation was thought to be most consistent with CAR T cell therapy-associated polyradiculomyelitis. Hence, on Day +64, the patient was treated with dexamethasone (20 mg) and continued at 10 mg daily for six additional days with no immediate significant improvement in the symptoms. On Day +76, treatment was switched to intravenous immunoglobulin (IVIG) at 1 g/kg for two doses every two weeks for three cycles, resulting in significant improvement in the paresthesia, weakness, and gait. She no longer required a cane to ambulate, and IVIG was decreased to a monthly schedule.

Four weeks later, on Day +156, the patient developed severe acute pain affecting her trunk and proximal extremities requiring hospital admission for pain management. She described stiffness, muscle spasms, involuntary extremity movements, and transient inability to move after rapid standing. A neurological exam at this time showed normal leg strength bilaterally and resolution of hyperreflexia. A repeat total spine MRI was unchanged from before. Serum analysis revealed the presence of glycine receptor alpha1 subunit (GlyR) and glutamic acid decarboxylase 65 (GAD65) antibodies, at a concentration of 0.14 nmol/L (reference range ≤0.02 nmol/L). Repeat EMG showed denervation of lumbosacral muscles with resolution of active denervation in the lower extremities. She started a pain management regimen which included duloxetine, gabapentin, baclofen, and controlled-release morphine sulfate. This was around Day +165 after CAR T cell therapy and a *BCR-ABL1* gene fusion was detected at this time prompting the resumption of ponatinib. Given the initial improvement she had with IVIG, the patient was restarted on IVIG at 1 g/kg every two weeks with the addition of weekly rituximab at 375 mg/m^2^. The rituximab was discontinued after three doses because of severe thrombocytopenia, and IVIG was also discontinued after four doses because of persistent pain and thrombocytopenia. Throughout this period, the patient had evidence of B-cell aplasia as demonstrated by the absence of B cells by flow cytometry.

At Day +204, after only a brief period of symptom improvement, the patient redeveloped pain and stiffness in the neck, shoulders, bilateral upper and lower extremities interfering with her daily activities and ability to sleep. It was decided not to do plasma exchange given the possibility of removing persistent CAR T cells. Therefore, she was treated with two doses of obinutuzumab on Days +302 and +316 with significant improvement in symptoms. Although the plan was to dose obinutuzumab every six months, the patient had a flare of symptoms on Day +388 and received an extra dose earlier than expected. The patient continues to require analgesics and has mild upper body stiffness, but has been able to resume activities of daily living. Ponatinib was switched to asciminib because of recurrent thrombocytopenia, and her disease remains in MRD negative remission.

## Discussion

CAR T cell therapy has arisen as a highly effective therapy in hematologic malignancies with high response rates. More than 60 % of patients receiving CAR T cell therapy may experience neurologic toxicity with variable degrees of severity.^1^ Patients with ALL are described as having a high rate of ICANS, with a reported incidence of 40–62 %.^2^, ^3^, ^4^ ICANS can manifest as delirium, headache, language disturbance, tremor, transient focal weakness, behavioral disturbances, ataxia, peripheral neuropathy, visual changes, generalized weakness, seizures, and acute cerebral edema.^5^, ^6^, ^7^ More severe cases of ICANS have been seen with severe CRS, suggesting a possible overlap between these two syndromes.^3^^,^^4^^,^^8^ Proposed mechanisms of ICANS include endothelial activation and the upregulation of proinflammatory cytokines in the central nervous system.^9^, ^10^, ^11^ Ideal management of these patients and long-term effects of ICANS are areas of active investigation.

Our patient’s course has several unique aspects. On Day +6 after receiving CAR T cell therapy, she developed progressive severe bilateral lower extremity burning pain followed by paraparesis, symptoms not typically reported with this therapy. She was ultimately treated for CAR T cell therapy-related lumbosacral polyradiculopathy treated with corticosteroids and IVIG to which she had a positive response. However, after initial improvement, her symptoms recurred with severe back pain, and stiffness of her neck, shoulders and bilateral upper and lower extremities. Additional testing demonstrated GlyR antibodies within the patient’s serum. Low titer GAD65 antibodies were also present, which have been described in patients with GlyR antibody syndromes.

GlyR autoantibodies have been described in patients with SPS, particularly the subtype progressive encephalomyelitis with rigidity and myoclonus (PERM). SPS (formerly stiff man syndrome) is a rare and disabling disorder characterized by truncal stiffness, muscle spasms, and impaired gait. Additional features of active denervation on EMG and CSF pleocytosis are atypical for SPS, and suggest possible PERM. However, the patient did not have other specific symptoms of PERM such as encephalopathy, brainstem features, or autonomic dysfunction.^12^ Carvajal-González et al.^13^ found that nine out of 52 cases of GlyR antibody syndrome had a diagnosis of malignancy at some point in their lives. The literature suggests a paraneoplastic incidence rate of 20 %, including in patients with thymoma, Hodgkin’s lymphoma and cancers of the lung, kidney and breast. GlyR autoantibodies have been reported in patients with underlying malignancies including thymoma, B-cell lymphoma, Hodgkin’s lymphoma, breast cancer, and small cell lung cancer.^14^, ^15^, ^16^ IVIGs, plasma exchange and B-cell depletion using rituximab have been used to suppress the presumed causative antibody-mediated process.

The temporal relationship of our patient’s symptoms with CAR T cell therapy point strongly towards a CAR T cell-mediated etiology, although the mechanism remains uncertain. The positive serological findings for GAD65 and glycine receptor antibodies could support a paraneoplastic etiology, such *As SPS*, however persistent remission and the timing of the emergence of symptoms shortly after CAR T cell therapy suggest an association with the therapy. A complication of CAR T cell therapy is B-cell aplasia and hypogammaglobulinemia and therefore the therapy has been under investigation for the treatment of autoimmune diseases. Hence, it is counter-intuitive that an autoimmune phenomenon develops post-treatment. It is possible that CAR T cells were exposed to certain antigens that led to antibody formation prior to development of B-cell aplasia, or that the threshold for the development of symptoms in the presence of pre-existing antibodies is lower. The etiology may also be cell-mediated secondary to immune dysregulation, cytokine release syndrome, and CAR T cell proliferation and persistence. In these scenarios, T cells are responsible for the immune response with subsequent activation of phagocytes, cytotoxic T cells and cytokine production.

We believe that CAR T cell-related autoimmune/inflammatory phenomena are a worthwhile consideration for future patients who may have unexplained neurological symptoms, especially as the long-term side effects remain uncertain. Importantly, treatment with corticosteroids and IVIG can be effective in such cases.

## Conflicts of interest

The authors declare no conflicts of interest
